# Improved metabolic control using glucose monitoring systems leads to improvement in vibration perception thresholds in type 1 diabetes patients

**DOI:** 10.1007/s00592-019-01450-2

**Published:** 2019-11-08

**Authors:** Lars B. Dahlin, Targ Elgzyri, Magnus Löndahl, Linnéa Ekman, Eero Lindholm

**Affiliations:** 1 Department of Translational Medicine - Hand Surgery, Lund University, Malmö, Sweden; 2 Department of Hand Surgery, Skåne University Hospital, Malmö, Sweden; 3 Department of Clinical Sciences, Endocrinology, Lund University, Jan Waldenströmsgata 24, 205 02 Malmö, Sweden; 4 Department of Clinical Sciences, Endocrinology, Lund University, Lund, Sweden

**Keywords:** Diabetes, Neuropathy, Continuous glucose monitoring, Flash glucose monitoring, Vibration perception thresholds, HbA_1c_

## Abstract

**Aims:**

Few studies have examined how improved metabolic control might influence vibration perception thresholds (VPTs). The aim of this study was to evaluate if improved HbA_1c_ can influence vibration thresholds in adults with type 1 diabetes (T1DM).

**Methods:**

VPTs were investigated at six frequencies (4–125 Hz) using VibroSense Meter in the sole of the foot at two occasions in 159 T1DM patients, at the heads of the first and fifth metatarsal bones, i.e. MTH1 and MTH5, respectively. The participants were divided into three groups: group A: HbA_1c_ improved by more than 1 mmol/mol (*n* = 95), group B: HbA_1c_ deteriorated by more than 1 mmol/mol (*n* = 48) and group C: HbA_1c_ unchanged (± 1 mmol/mol) (*n* = 16) compared to baseline.

**Results:**

In group A, the mean *z*-score, reflecting the combined effect of all VPTs, improved being lower at the follow-up than at the baseline [0.2 (− 0.3 to 1.2) vs. −0.1 (− 0.7 to 0.8), *p* = 0.00002]. VPTs improved at 4 and 64 Hz at both MTH1 (metatarsal head 1) and MTH5. The VPTs at 125 Hz frequency improved at MTH5, but not at MTH1. No significant differences were seen in group B or group C.

**Conclusions:**

Lower HbA_1c_ and lower VPTs in T1DM patients were associated with improved VPT, suggesting a reversible effect on nerve function by improved metabolic control.

## Background

Diabetic neuropathies are common, and the most common form of neuropathy is distal symmetric polyneuropathy (DSPN). It is estimated that prevalence of DSPN in patients with type 1 diabetes (T1DM) is at least 20% after 20 years of disease duration [1]. Although DSPN is often considered irreversible, some studies suggest that both peripheral neuropathy and autonomous neuropathy can be reversed in specific cases [2–5], even if structural changes are present with degeneration and regenerative clusters [6]. Normalization of blood glucose by pancreas transplantation can improve nerve function in T1DM patients with DSPN [2], although severe nerve fibre loss seems to be irreversible [7]. In obese patients with type 2 diabetes (T2DM), gastric bypass leads to improvement in both Neuropathy Symptom Score and Neuropathy Deficit Score 6 months after surgery and this improvement seems to be at least partially independent of the improvement in HbA_1c_ [5]. Lifestyle interventions in patients with impaired glucose tolerance resulted in improvement in intraepidermal nerve fibre density (i.e. small diameter nerve fibres) despite rather modest improvements in metabolic markers [8]. Similar data have been obtained regarding autonomic neuropathy as improvement in metabolic control can improve heart rate variability in T1DM patients with early cardiac autonomic neuropathy (CAN), while it continued to deteriorate in patients with more advanced CAN [3].

Studies on the effects of improved metabolic control on DSPN in T1DM and T2DM are scarce. A recent study in 25 patients with diabetes investigated nerve fibre density measured by corneal confocal microscopy in both T1DM and T2DM during 24-month follow-up. Increase in nerve fibre density was significantly associated with the reduction in HbA_1c_ from baseline to follow-up [4].

The effect of improved metabolic control on vibration perception thresholds (VPTs) has been so far mostly disappointing. A recent review by Callaghan et al. concluded that randomized control trials on enhanced glucose control in T1DM patients show effect on some measured parameters (different nerve conduction velocities), but no improvement in VPTs although a meta-analysis showed a marginally significant difference in VPTs in favour of intensified glucose control [9]. The largest study followed 102 patients (48 with intensified treatment/54 standard insulin treatment) for 7.5 years [10]. The tibial, peroneal and sural nerve velocities showed less deterioration in the intensive treatment group compared to the standard treatment. However, no difference in VPTs, measured by using a biothesiometer, was seen. A small study in Japanese patients with T2DM could, however, show a positive effect of enhanced short-term (20 days) glycaemic control, which resulted in improved vibration sensation measured with 64-Hz Rydel–Seiffer tuning fork [11]. None of the 31 patients had neuropathic symptoms, and the duration of diabetes was rather short (10 years), suggesting that patients that were studied did not have an advanced diabetic neuropathy. A recent study on newly diagnosed patients with T2DM could demonstrate that intensive treatment leading to normalization of HbA_1c_ (from 81.4 mmol/mol (9.6%) to 41.4 mmol/mol (6.0%) led to long-term improvement in vibration perception thresholds measured by the biothesiometer (VPT frequency 120 Hz) [12].

Multifrequency vibrometry is a novel method for assessment of neuropathy. VPTs are measured at six frequencies (4, 8, 16, 32, 64 and 125 Hz) at the sole of the foot. We have recently shown that VPTs at low frequencies (4 to 8 Hz) are a better indicator for the risk of developing diabetic foot ulcers than higher frequencies (like 64 or 125 Hz). Low frequencies were also associated with certain neuropathic symptoms like gait or balance problems or weakness of the foot [13].

The aim of this study was to examine long-term effects of metabolic control on VPTs measured by multifrequency vibrometer.

## Research design and methods

### Patients and methods

In total, 215 T1DM patients, attending the department of Endocrinology, Skåne University Hospital, Malmö, Sweden, were examined twice during the period between 2015 and 2018. Patients, who had HbA_1c_ measured within 3 months at the time of the inclusion and during the follow-up visit (*N* = 161), were selected. One patient having hypoglycaemia at the time of the visit was excluded as well as one patient receiving chemotherapy since these conditions could affect the VPTs. Finally, 159 patients (73 males and 86 females) were included. Patients were examined the second time after 1–3 (mean 1.8 ± 0.5) years. Vibration perception thresholds were measured at MTH1 and MTH5 with a standard VibroSense Meter (VibroSense Dynamics, Malmö, Sweden). The method has been described previously in detail [13, 14]. In the statistical analysis, only VPTs that were measured at the right foot were used.

At the same time (spring 2015), flash glucose monitoring (FGM) was introduced at the clinic. At follow-up visit, 128 patients had either FGM (*n* = 109) or other types of continuous glucose monitoring system (CGM) (*n* = 19). During the study period, one patient had CGM at inclusion, but discontinued it shortly after the basal visit and 30 patients did only use traditional self-monitoring of blood glucose (SMBG) by finger prick.

HbA_1c_ was measured using either Capillarys 3 TERA Haemoglobin A1c Kit-program (Sebia Benelux SCS, Belgium) with CV of 3% or Afinion™ AS100 Analyzer (Abbott Laboratories, Chicago, Illinois, USA) with CV 4% at low HbA_1c_ (mean 43.6 mmol/mol (6.1%)) and 2.6% at high HbA_1c_ (mean 65.8 mmol/mol (8.2%)). External quality assessment of HbA_1c_ measurements is organized by the non-profit organization Equalis [15], and both methods are traceable to the International Federation of Clinical Chemistry and Laboratory Medicine (IFCC) Ref. [16]. Afinion™ method was used for 5 measurements at the baseline and 45 times at the follow-up. The HbA_1c_ and the change in HbA_1c_ were similar regardless of which method was used (data not shown).

To evaluate the effects of metabolic control on VPTs, patients were divided into three different groups: group A: those who had improved their metabolic control more than 1 mmol/mol, group B: patients whose HbA_1c_ had increased more than 1 mmol/mol and group C: patients whose HbA_1c_ was similar (± 1 mmol/mol) compared to baseline.

### Statistical analysis

Data on normally distributed values are presented as mean ± standard deviation (SD). Vibration thresholds, diabetes duration and S-triglycerides were not normally distributed and are given as median and 25th and 75th percentile. Because VPTs increase with increasing frequency, a *z*-score for each frequency was calculated using the formula *z* = (*x*−*μ*)/*σ*, where *x* is the VPT value, while *μ* and *σ* are the age- and gender-specific mean and standard deviation, respectively, for a normal population. A mean *z*-score for each observation was then calculated from all individual *z*-scores and gave an estimate of all VPT frequencies together. Wilcoxon signed-rank test was used to compare VPTs at the baseline and at follow-up visit. To correct for multiple comparisons, the significance level was defined as *p* = 0.004 (0.05/13). IBM SPSS Statistics (Statistical Package for the Social Sciences, SPSS Inc., Chicago, Il, USA) version 25 was used for all statistical analyses.

## Results

In total, 95 patients had lower HbA_1c_ at the follow-up visit than at baseline (group A) (Table 1). HbA_1c_ deteriorated from baseline in 48 patients (group B). HbA_1c_ changed 1 mmol/mol or less in 16 patients (group C). Baseline HbA_1c_ was higher in group A as compared to group B (66.3 ± 13.5 vs. 56.2 ± 10.3 mmol/mol, *p* = 0.00001), but lower at follow-up (56.5 ± 11.6 vs. 62.8 ± 11.7 mmol/mol, *p* = 0.003). HbA_1c_ in group C did not differ from group A or B, neither at baseline nor at follow-up. Patients, who switched from SMBG to either FGM or CGM during the study period, reduced their HbA_1c_ significantly compared to those who continued with SMBG during the whole study period [6.1 ± 9.5 vs. 0.7 ± 8.0 mmol/mol (0.6% vs. 0.1%), *p* = 0.01]. The serum lipid values did not change between visits.

**Table 1:** Patient characteristics at baseline and change in HbA_1c_ from baseline categorized according to change in the HbA_1c_

	Group A	Group B	Group C
N (M/F)	95 (41/54)	48 (23/25)	16 (9/7)
Age (years)	47.2 ± 15.7	46.8 ± 15.5	50.8 ± 17.1
Age at onset (years)	25.3 ± 15.6	25.6 ± 13.4	23.4 ± 13.3
Duration (years)	16.9 [7.9–34.4]	19.6 [9.0–31.5]	27.5 [17.7–36.0]
Height (cm)	172.1 ± 11.2	172.8 ± 15.3	174.4 ± 9.6
Weight (kg)	77.4 ± 16.3	73.6 ± 12.3	79.3 ± 15.7
HbA_1c_ (mmol/mol)/(%)	66.3 ± 13.5 (8.2 ± 1.2)	56.2 ± 10.3 (7.3 ± 0.9)^a^	61.3 ± 11.5 (7.8 ± 1.1)
ΔHbA1c mmol/mol)/(%)	− 9.8 ± 8.6 (− 0.9 ± 0.8)	6.6 ± 4.3 (0.6 ± 0.4)^a^	0.3 ± 0.8 (0.0 ± 0.1)^a^

Wilcoxon sign rank test was used for S-triglycerides and duration and paired *t* test for other variables. Group A: HbA_1c_ improved by more than 1 mmol/mol, group B: HbA_1c_ deteriorated by more than 1 mmol/mol, group C: HbA_1c_ unchanged (± 1 mmol/mol) compared to baseline. ^a^*p* < 0.0001 compared to group A

Age, age at onset, diabetes duration or serum lipid values did not differ between the groups. The mode of insulin delivery was similar in groups A and B (24.2% vs. 29.2%, respectively). Insulin pump was used more frequently in group C than in other two groups (62.5%).

Table 2 shows the VPTs for the different groups based on the change in HbA_1c_. In group A, vibration perception thresholds were significantly improved at the follow-up visit [*z*-score 0.2 (− 0.3 to 1.2) vs. − 0.1 (− 0.7 to 0.8), *p* = 0.00002]. No significant differences in the mean *z*-scores were seen in patients with deteriorated or unchanged metabolic control.

**Table 2:** Comparison of change in vibration perception thresholds at baseline versus follow-up in different categories of HbA_1c_ change

Frequency	Group A	Group B	Group C
MTH1 4 Hz (dB)	3.1 [− 2.3 to 7.4]^a^	0.0 [− 5.5 to 3.8]	3.0 [0.0 to 5.2]
MTH1 8 Hz (dB)	1.7 [− 1.9 to 6.6]	0.0 [− 3.3 to 2.3]	1.0 [− 8.1 to 3.2]
MTH1 16 Hz (dB)	0.1 [− 4.0 to 6.1]	− 1.1 [− 5.5 to 3.7]	− 3.1 [− 7.8 to 4.4]
MTH1 32 Hz (dB)	2.2 [− 3.2 to 6.4]	1.2 [− 2.0 to 4.5]	− 2.5 [− 7.7 to 5.4]
MTH1 64 Hz (dB)	4.7 [1.1 to 10.1]^b^	2.2 [− 3.8 to 6.9]	− 0.3 [− 10.6 to 4.7]
MTH1 125 Hz (dB)	1.8 [− 2.8 to 7.0]	0.0 [− 4.0 to 4.8]	− 0.7 [− 4.2 to 9.2]
MTH5 4 Hz (dB)	4.0 [0.0 to 8.4]^c^	2.7 [− 2.4 to 7.7]	3.8 [− 2.9 to 7.3]
MTH5 8 Hz (dB)	2.7 [− 1.9 to 7.4]	1.6 [− 3.6 to 6.7]	1.4 [− 2.2 to 9.4]
MTH5 16 Hz (dB)	1.6 [− 3.7 to 6.5]	2.1 [− 4.4 to 7.7]	2.3 [− 0.8 to 8.4]
MTH5 32 Hz (dB)	2.1 [− 3.2 to 7.2]	2.5 [− 3.9 to 6.9]	1.6 [− 2.4 to 5.3]
MTH5 64 Hz (dB)	5.3 [− 1.6 to 13.0]^c^	3.1 [− 1.8 to 7.4]	1.5 [0.6 to 7.8]
MTH5 125 Hz (dB)	4.4 [− 2.1 to 9.9]^c^	2.1 [− 6.9 to 7.3]	0.7 [− 2.8 to 13.3]
*z*-score (all)	− 0.33 [− 0.02 to 0.72]^c^	− 0.06 [− 0.18 to 0.47]	− 0.14 [− 0.22 to 0.44]

Wilcoxon sign rank test. *p* < 0.004 is considered as significant after correction for multiple (13) comparisons. Group A: HbA_1c_ improved by more than 1 mmol/mol, group B: HbA_1c_ deteriorated by more than 1 mmol/mol, group C: HbA_1c_ unchanged (± 1 mmol/mol) compared to baseline. Values are change in VPTs [25th–75th percentile]. ^a^*p* < 0.004; ^b^*p* < 0.001; ^c^*p* < 0.0001

In group A, VPTs at the 4 Hz frequency were significantly lower both at MTH1 [96.2 (91.7–108.7) vs. 95.1 (89.9–102.4) dB, *p* = 0.003] and MTH5 [97.8 (92.5–106.8) vs. 94.2 (90.2–101.9) dB, *p* = 0.000003] at the follow-up visit compared to baseline. VPTs at 64 Hz were also significantly lower at follow-up at both localizations [128.6 (119.0–139.7) vs. 125.6 (114.9–135.8) dB, *p* = 0.0002 and 127.9 (120.3–138.0) vs. 126.4 (117.6–135.1) dB, *p* = 0.00006, respectively]. VPTs at the 125 Hz frequency were lower at follow-up in group A at MTH5, but not at MTH1. In group A, there were also differences in VPTs at 8 and 32 Hz frequencies (both MTH1 and MTH5), but these were not statistically significant when corrected for multiple comparisons (13).

In group B, the VPTs at 64 Hz frequency at MTH5 were lower at follow-up compared to baseline, although the difference was not significant when considering multiple comparisons. In patients with unchanged HbA_1c_ (group C), both 16 and 64 Hz were lower at the follow-up; however, the difference was not significant after correction for multiple comparison.

In group A, 10 patients used CGM and 15 patients used SMBG only. At follow-up, both of these subgroups had lower VPTs measured at MTH5 for the 4 Hz frequency than at baseline [CGM 96.5 (92.1–103.2) dB vs. 92.6 (82.6–1001.2) dB, *p* = 0.02; SMG 97.6 (94.7–106.6) dB vs. 94.1 (90.8–101.5) dB, *p* = 0.03, baseline vs. follow-up, respectively].

In a subgroup of patients (*N* = 10) in group A with long duration of diabetes (> 20 years) and severely impaired VPTs at baseline (mean *z*-score > 2.0), the 4 Hz frequency improved from baseline to follow-up. VPTs for 4 Hz were at MTH1 113.0 (108.8–115.0) dB vs. 108.5 (102.2–115.0) dB, *p* = 0.02 and at MTH5 115.0 (115.0–115.0) dB versus 110.0 (102.3–115.0) dB, *p* = 0.04. For 64 Hz, the VPTs were at MTH1 150.1 (145.9–156.7) dB versus 146.5 (141.9–155.5) dB, *p* = 0.02 and at MTH5 155.4 (147.4–156.5) dB versus 143.9 (139.7–149.1) dB, *p* = 0.03, respectively.

## Discussion

The present study has shown that improved metabolic control in patients with T1DM is associated with improved vibrotactile sense observed by lower vibration perception thresholds at low frequencies, i.e. at 4 and 64 Hz. Previous studies in T1DM patients have shown improvement in conduction velocities of the peroneal, median and ulnar nerves, but not in VPTs [9]. In contrast, two other studies, including people with T2DM [11, 12], have shown improvement in vibration perception after intensive glucose control, i.e. one in patients with newly diagnosed diabetes and one with diabetes duration up to 10 years. The improvements in HbA_1c_ in these studies of patients with T2DM were large [19.6 and 41.4 mmol/mol (1.8% and 4.6%), respectively] compared to a rather modest improvement in our present study [9.8 mmol/mol (1.0%) in group A]. It is possible that patients with T1DM in earlier studies did not have sufficient decrease in HbA_1c_ or that their long diabetes duration was associated with more advanced structural changes that were not reversible. Another explanation could be the difference in the equipment and frequencies used to test VPTs. Thus, in the Stockholm Diabetes Intervention Study, with a diabetes duration similar to ours (intensive treatment group: 18 years and conventional treatment group: 16 years) [10], and with a larger HbA_1c_ reduction [− 25 mmol/mol (2, 4%)], the biothesiometer measuring VPTs at 100 Hz frequency was used.

Out of the six frequencies measured in our study, the 4 and 64 Hz frequencies showed improved VPTs regardless of the site, while the 125 Hz frequency measured at MTH1 did not show any difference. Our interpretation is that the 4 Hz frequency is more sensitive to changes in metabolic control than 125 Hz. Another possibility could be that the pressure against the skin varies when using a handheld instrument. An increase in pressure has been shown to give decreased VPTs [17]. In the VibroSense Meter, the contact pressure of the probe against the skin is adjusted prior to the measurement to a force of approximately 0.2 N, and if the pressure would change, it will be corrected during the test.

A long duration of diabetes or an advanced peripheral neuropathy, e.g. more advanced structural changes, already at start could be possible explanations why earlier studies did not show significant improvement in VPTs in patients with T1DM despite the improved metabolic control. In the present study, however, VPTs at 4 Hz and 64 Hz improved with improving HbA_1c_ even in patients with a long duration of diabetes and severely impaired VPTs at the baseline.

One limitation of this study is that it is not a randomized trial, but an observational study. The introduction of the FGM and the CGM systems coincided with the present study and was accompanied by larger reductions in HbA_1c_ than was anticipated, which resulted in a larger group of individuals with improved metabolic control and a smaller group of individuals with unchanged or impaired HbA_1c_.

Both CGM [18] and FGM [19] decrease glucose variability, and this could be a possible explanation for the improved VPTs. We did not record any measures of glucose variability and therefore cannot answer the question what role it may play. Only 15 patients improved their metabolic control using SMBG. However, the difference in 4 Hz frequency was still significant, suggesting that metabolic control itself is important.

We did not measure plasma glucose at the time of the investigation. It has been shown that very short-term hyperglycaemia does not influence VPTs [20] even though large improvements in metabolic control during 20 days influenced vibration sensation [11].

In summary, we show that signs of peripheral neuropathy, based on measurements of VPTs, in patients with T1DM may be reversible if metabolic control is improved. As a novel finding, we demonstrate that VibroSense Meter can monitor changes in VPTs after improvement in metabolic control. This can further motivate patients with T1DM to continue to improve their HbA_1c_.
